# Efficacy comparison of five antidepressants in treating anxiety and depression in cancer and non-cancer patients

**DOI:** 10.3389/fnins.2024.1485179

**Published:** 2024-10-30

**Authors:** Kuan Zhao, Youyang Wang, Qun Liu, Ze Yu, Wei Feng

**Affiliations:** ^1^Department of Psychological Medicine, Fudan University Shanghai Cancer Center, Shanghai, China; ^2^Department of Oncology, Shanghai Medical College, Fudan University, Shanghai, China

**Keywords:** cancer, anxiety, depression, antidepressants, efficacy

## Abstract

**Introduction:**

Cancer patients have a heightened susceptibility to anxiety and depressive disorders, which significantly impact the effectiveness of cancer treatments and long-term quality of life. This study aimed to compare the efficacy of different antidepressants in cancer and non-cancer patients.

**Methods:**

A total of 610 patients diagnosed with depressive episodes and/or anxiety disorders were retrospectively included and divided into a cancer group and a non-cancer control group. Antidepressants used included escitalopram, duloxetine, sertraline, venlafaxine, and vortioxetine, combined with trazodone or not. The Patient Health Questionnaire-9 (PHQ-9) and the Generalized Anxiety Disorder Questionnaire-7 (GAD-7) scores were used to evaluate the efficacy after 4 weeks and 8 weeks of systematic antidepressants treatment.

**Results:**

Compared to the non-cancer group, the cancer group had higher proportions of females, older individuals, and patients with poor sleep quality, while reporting fewer somatic symptoms at baseline (all *p* < 0.05). PHQ-9 and GAD-7 scores in cancer patients treated with antidepressants were significantly lower than baseline at week 4 and week 8 (all *p* < 0.05). The sertraline group demonstrated significantly less improvement in GAD-7 scores at week 4 and in both GAD-7 and PHQ-9 scores at week 8 compared to the escitalopram group, while duloxetine, venlafaxine, and vortioxetine showed comparable efficacy to escitalopram. Antidepressants combined with trazodone showed significant improvement in PHQ-9 scores at week 4 compared to those without trazodone. The gynecological cancer group showed significantly more improvement in GAD-7 and PHQ-9 scores at week 4 and 8 compared to breast cancer patients.

**Conclusion:**

Antidepressant treatment in cancer patients with anxiety and depression is as effective as in non-cancer patients. The efficacy of escitalopram is comparable to duloxetine, venlafaxine, and vortioxetine, all of which outperformed sertraline in cancer patients.

## 1 Introduction

An estimated 19.3 million new cancer cases and nearly 10 million cancer-related deaths were recorded globally in 2020, highlighting urgent concerns in global cancer management (Sung et al., 2021; Qiu et al., 2021). Cancer patients were more susceptible to major depressive disorder (MDD) and anxiety symptoms than non-cancer patients (Vehling et al., 2022; Mitchell et al., 2011). However, whether the efficacy of common antidepressants used in cancer patients has seldom been investigated.

Cancer patients usually suffer from physical and phycological pathologies leading to anxiety and depression, such as immune dysregulation, increased inflammation, anhedonia due to altered cortisol levels, mental trauma from a sudden positive cancer diagnosis, and physical symptoms caused by the side effects of chemotherapeutic treatments. Emotional disorders are also attributed to patients’ fear of death, unpleasant changes in the local environment, reduced social interactions, psychological stress, and persistent depression (Chan et al., 2023; Fetcho et al., 2023). The risk of self-injury in cancer patients with untreated emotional disorders has been increased significantly, affecting their quality of life (QoL) (Heinrich et al., 2023; Grassi, 2020). Therefore, the management of anxiety and depression is important in improving patients’ adaptation and well-being.

Although antidepressants are commonly prescribed and established as effective treatments for anxiety and depression in non-cancer patients, their efficacy in cancer patients is not well-defined. Furthermore, cancer patients usually have aberrant inflammatory alternation, leading to decreased psychomotor speed, which has been associated with poor antidepressant treatment response (Goldsmith et al., 2016). To address these questions, here we aim to compare the efficacy of antidepressants in cancer and non-cancer patients, and also to evaluate the efficacy of escitalopram, duloxetine, sertraline, venlafaxine, and vortioxetine, combined with trazodone or not among cancer patients.

## 2 Materials and methods

### 2.1 Subjects

The data for this study were collected from the database of patients visiting the Department of Psychology at Fudan University Shanghai Cancer Center between July 2021 and September 2023. Ethics approval was obtained from Medical Ethics Committee, Fudan University Shanghai Cancer Center. The eligibility criteria for patients were as follows: (1) age ≥ 18 years; (2) diagnosed with “depressive episode” and/or “anxiety disorder” according to ICD-10; (3) completed psychological assessments at the initial visit; and (4) received antidepressant treatment. The exclusion criteria included: (1) inability to understand or execute relevant assessments; (2) comorbid schizophrenia, bipolar disorder, epilepsy, cognitive impairment, or other psychiatric conditions; and (3) continuing medications for pre-existing antidepressants for more than one week or were concurrently using antipsychotics, mood stabilizers, or other psychotropic medications. Following the screening procedure, a total of 610 patients were enrolled in this study.

### 2.2 Behavioral assessments

**FIGURE 1:**
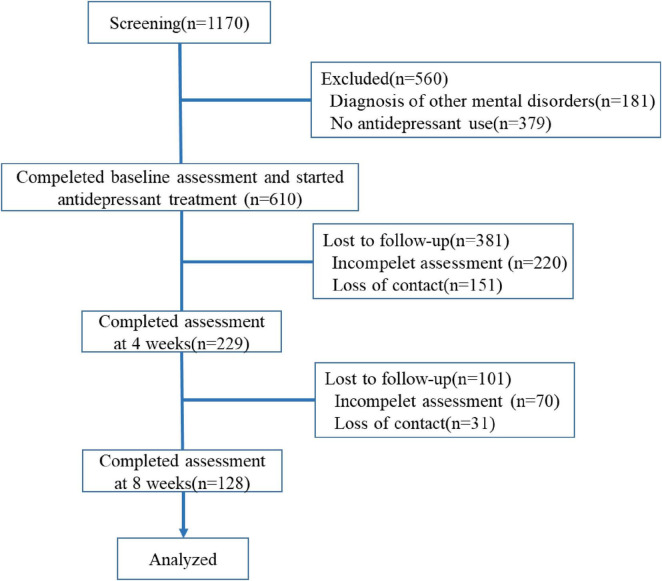
Study flow chart.

For the behavioral evaluations, we collected patients’ demographics such as gender and age, and clinical characteristics, including the cancer type, staging, past/ongoing anti-cancer treatments, history of psychiatric diagnosis (if any), type of antidepressant used, and scores on 9-item Patient Health Questionnaire (PHQ-9), PHQ-15, GAD-7, Pittsburgh Sleep Quality Index (PSQI), and Visual Analog Scale (VAS) for pain. Follow-up assessments were conducted at 4-week and 8-week post-enrollment time points, with evaluations of PHQ-9 and GAD-7 scores (see Figure 1 for details). PHQ-9 is a self-reporting scale used to assess the severity of depression in patients. It consists of 9 items, with each item scoring on a Likert scale ranging from 0 to 3, resulting in a total score of 0 to 27. A score of ≥ 8 in cancer patients is considered clinically significant for depression (Manea et al., 2012). GAD-7 is another self-reporting scale designed to evaluate the severity of anxiety in patients. It comprises 7 items, each scoring on a Likert scale from 0 to 3, yielding a total score of 0 to 21. A score of ≥ 10 in cancer patients indicates clinically significant anxiety (Esser et al., 2018). The PHQ-15 is used to assess the severity of somatic symptoms in patients. It includes 15 somatic symptom clusters, including some of the common symptoms such as fatigue, pain, and various gastrointestinal disorders, as observed in cancer patients (Kroenke et al., 2002). PSQI is used to evaluate the patient’s sleep quality over one month. A score of > 5 suggests poor sleep quality, which has been validated in cancer patients (Momayyezi et al., 2021). The VAS is measured on a 0–10 scale and assesses the current level of pain experienced by the patient. Its reliability and reproducibility have been validated in cancer patients (Li et al., 2021).

### 2.3 Antidepressants

Patients were divided into antidepressants groups according to their prescription. Commonly used antidepressants were compared in this study, including selective serotonin reuptake inhibitors (SSRIs) like escitalopram and sertraline, serotonin-norepinephrine reuptake inhibitors (SNRIs) such as venlafaxine and duloxetine, and vortioxetine, which has multiple effects on serotonin receptors. Escitalopram, in particular, was used as the control drug due to its relatively high efficacy and lower incidence of side effects compared to other antidepressants in general population (Yin et al., 2023).

### 2.4 Cancer type categories and current cancer stage

In the cancer group, patients were categorized as follows: breast cancer (132), digestive system cancer (45), endocrine organ cancer (27), gynecological cancer (34), head and neck cancer (13), hematologic malignancy (4), lung cancer (19), soft tissue cancer (4), and male reproductive/urinary system cancer (10). Treatment modalities included surgery (222), radiotherapy (81), chemotherapy (142), immunotherapy (9), targeted therapy (34), and endocrine therapy (49). Patients were further grouped based on their current cancer stage: undergoing treatment (202), in remission (60), and cancer reoccurring (20).

### 2.5 Statistical analysis

All statistical analyses were performed using R (version 4.3.0). Quantitative data were expressed as mean ± standard deviation (SD) or median. Patients were divided into two groups – non-cancer and cancer. The t-test was used to compare differences in quantitative data between the two groups. A non-parametric test was conducted for non-normally distributed data. The chi-square (χ^2^) test was used to compare differences in qualitative data between the groups. The population of patients with intention-to-treat was analyzed in this study, and missing values were adjusted. A mixed linear model (LMM) was used to analyze repeatedly measured data, using maximum likelihood estimation to estimate model parameters. This approach allows LMM to utilize available observed data for estimation, even in the presence of missing values. Subgroup analysis was conducted for patients in the cancer group. The Akaike Information Criterion (AIC) was used to measure the goodness of fit of a statistical model; a smaller AIC value indicates a better model fit. For linear mixed models, different models may have different fixed effects and random effects structures, and AIC provides a unified standard to evaluate and compare these models (Liu et al., 2024). A *p*-value of < 0.05 indicated the statistical significance of the result.

## 3 Results

### 3.1 Baseline characteristics

A total of 610 patients were included in this study, with 288 patients in the cancer group and 322 patients in the non-cancer group (Table 1). The age and gender were significantly different in cancer and non-cancer groups.

**TABLE 1:** Comparison of clinical characteristics between cancer group and non-cancer group at baseline.

		Cancer group (288)	Non-cancer group (322)	t/χ^2^	*p*
Gender*	Male	46 (33.86%)	90 (66.14%)	12.60	<0.01
	Female	242 (51.15%)	232 (48.85%)		
Age*		50 ± 12	38 ± 16	94.43	<0.01
Psychiatric diagnosis *	Depressive episode	22 (45.83%)	58 (54.17%)	14.44	<0.01
	Anxiety disorders	94 (51.14%)	90 (48.86%)		
	Comorbid depression and anxiety	172 (49.72%)	174 (50.28%)		
Episode Type	First	244 (48.70%)	257 (51.30%)	2.17	0.14
	Relapse	44 (40.37%)	65 (59.63%)		
Antidepressants* (categories and dose)				**36.50**	**<0.01**
	Duloxetine	15 (65.20%)	8 (34.80%)	−1.14	0.27
	Dose(mg)	52.50 ± 14.88	61.33 ± 22.00		
	Escitalopram	246 (54.10%)	209 (45.90%)	0.27	0.79
	Dose(mg)	14.59 ± 3.61	14.50 ± 3.87		
	Sertraline	12 (21.14%)	45 (78.86%)	−1.20	0.24
	Dose(mg)	75.00 ± 33.71	88.89 ± 41.48		
	Venlafaxine	20(32.25%)	42(67.75%)	−1.58	0.12
	Dose(mg)	116.25 ± 61.92	142.86 ± 61.56		
	Vortioxetine	2 (15.40%)	11 (84.60%)	0.00	1.00
	Dose(mg)	15 ± 7.07	15 ± 4.47		
Combined trazodone*	Yes	195 (54.56%)	166 (46.43%)	16.43	<0.01
	No	93 (37.36%)	156 (62.64%)		
Scale assessment*	PHQ-9*	13.50 ± 5.85	14.70 ± 6.52	6.37	0.01
	GAD-7	12.97 ± 5.13	13.20 ± 5.33	0.40	0.52
	VAS	2.77 ± 3.64	2.87 ± 2.63	1.24	0.26
	PHQ-15*	11.16 ± 4.58	12.13 ± 5.37	4.54	0.03
	PSQI*	13.68 ± 4.51	12.16 ± 4.43	18.25	<0.01

*p*: *p*-values (2-tailed);

*p < 0.05; PHQ-9, Patient Health Questionnaire-9; GAD-7, Generalized Anxiety Disorder Questionnaire-7; VAS, Visual Analog Scale; PHQ-15, 15-item Patient Health Questionnaire; PSQI, Pittsburgh Sleep Quality Index.

Psychiatric diagnoses and antidepressant use differed significantly between the cancer and non-cancer groups (*p* < 0.05). In the cancer group, 45.83% had depressive episodes, 51.14% had anxiety disorders, and 49.72% had comorbid depression and anxiety, compared to 54.17%, 48.86%, and 50.28% in the non-cancer group. The proportion of first-episode patients was similar in the cancer group (48.70%) and the non-cancer group (51.30%), with no significant difference (*p* = 0.14). Cancer patients used escitalopram and duloxetine more frequently, while trazodone was mainly used in combination therapy in the cancer group (*p* < 0.01). The doses of antidepressants between the two groups were equivalent, with no statistically significant differences detected (*p* > 0.05). PHQ-9, PSQI and PHQ-15 scores score at baseline was significantly different in the cancer group within the non-cancer group (all *p* < 0.05). However, there was no significant difference in GAD-7 and VAS scores between the two groups (*p* > 0.05).

### 3.2 Correlation analysis of behavioral assessment results

**FIGURE 2:**
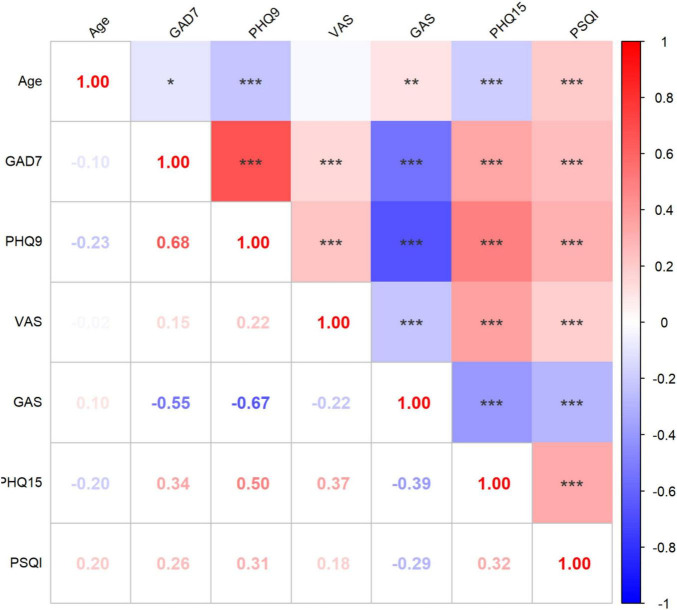
Correlation analysis of behavioral assessment results. *p < 0.05, **p < 0.01, ***p < 0.001; PHQ-9, Patient Health Questionnaire-9; GAD-7, Generalized Anxiety Disorder Questionnaire-7; VAS, Visual Analog Scale; PHQ-15, 15-item Patient Health Questionnaire; PSQI, Pittsburgh Sleep Quality Index. The gradient color error bar represents the Pearson’s *r*-value.

A correlation analysis was conducted to investigate relationships among the patient’s age and baseline behavioral assessment results (Figure 2). It demonstrated that elder patients tend to have significantly lower GAD-7, PHQ-9, and PHQ-15, but higher PSQI scores. Positive correlations were observed among all behavioral assessment scores with each other (*p* < 0.01).

### 3.3 Comparative efficacy of antidepressants in cancer and non-cancer patients

#### 3.3.1 Efficacy without adjusting confound factors

**FIGURE 3:**
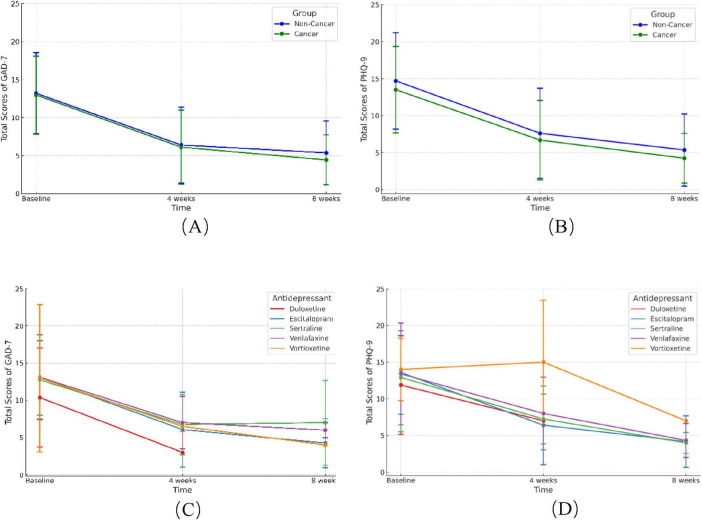
Change of GAD-7 and PHQ-9 scores after 4 and 8 weeks of antidepressants treatment. **(A,B)** Change of GAD-7 **(A)** and PHQ-9 **(B)** scores between cancer and non-cancer groups after 4 and 8 weeks of antidepressants. **(C,D)** Improvement of GAD-7 **(C)** and PHQ-9 **(D)** scores with five different antidepressants in cancer patients. GAD-7, Generalized Anxiety Disorder Questionnaire-7; PHQ-9, Patient Health Questionnaire-9.

The PHQ-9 and GAD-7 scores significantly reduced after 4- and 8-weeks treatment of antidepressants, while no significant differences were found in the improvement of scores between the cancer and non-cancer groups (Figures 3A, B, *p* > 0.05). We then compared the efficacy of five antidepressants in cancer patients (Figures 3C, D). After 4 weeks of treatment, PHQ-9 scores decreased in all antidepressant groups except the vortioxetine group, while GAD-7 scores decreased in all five groups. After 8 weeks, both PHQ-9 and GAD-7 scores significantly decreased in the escitalopram, sertraline, venlafaxine, and vortioxetine groups. The duloxetine group lacked sufficient data after 8 weeks due to patient loss to follow-up.

#### 3.3.2 Efficacy after adjusting confound factors

**(1) Impact of treatment duration**

To adjust various confounding factors and the interaction effects of repeated measurement, we employed linear mixed model to evaluate the efficacy of antidepressants (Oberg and Mahoney, 2007). It demonstrated that the duration of antidepressants treatment (4 or 8 weeks) had a significant effect on the outcome (*p* < 0.05). Among the various models tested, those with smallest AIC value was considered the best fit and demonstrated in Table 2 (*p* < 0.01). After adjusting baseline age, gender, anxiety or depression diagnoses, and episode type, the GAD-7 and PHQ-9 scores at the 4^th^ and 8^th^ weeks decreased significantly compared to baseline values (4^th^ week change of GAD-7: β = −7.46, SE = 0.0.52, *p* < 0.001; 4^th^ week change of PHQ-9: β = −7.45, SE = 0.58, *p* < 0.001; 8^th^ week change of GAD-7: β = −8.93, SE = 0.68, *p* < 0.001; 8^th^ week change of PHQ-9: β = −9.58, SE = 0.76, *p* < 0.001).

**TABLE 2:** Results of the mixed linear model of factors influencing PHQ-9 and GAD-7.

Variables	Categories	GAD-7 scores	PHQ-9 scores
Follow-up time	Baseline	0	0
	Week 4	−7.46 (0.52)***	−7.45 (0.58)***
	Week 8	−8.93 (0.68)***	−9.58 (0.76)***
Cancer status	Cancer	0	0
	Non-cancer	−0.04 (0.44)	0.22 (0.48)
Episode Type	First	0	0
	Relapse	0.27 (0.53)	0.45 (0.59)
Antidepressants	Escitalopram	0	0
	Duloxetine	−0.48 (1.05)	−1.05 (1.15)
	Sertraline	−1.04 (0.72)	0.28 (0.80)
	Venlafaxine	0.57 (0.67)	1.63 (0.74)*
	Vortioxetine	−1.36 (1.41)	1.02 (1.56)
Combined trazodone	Yes	0	0
	No	0.76 (0.67)*	−1.27 (0.46)**
Interaction effects (influencing factors × time)			
Week 4−baseline			
Cancer status group (non-cancer) × time		−0.31 (0.64)	−0.88 (0.72)
Episode Type(relapse) × time		0.36 (0.77)	0.77 (0.86)
Antidepressants group × time			
Escitalopram		0	0
Duloxetine		2.63 (2.28)	3.34 (2.54)
Sertraline		2.69 (1.07)*	1.68 (1.20)
Venlafaxine		1.44 (1.04)	0.99 (1.16)
Vortioxetine		3.40 (2.17)	3.27 (2.43)
Combined trazodone(no) × time		0.76 (0.67)	2.06 (0.74)**
Week 8 – baseline			
Cancer status group (non-cancer) × time		0.70 (0.81)	0.47(0.91)
Episode Type(relapse) × time		0.16 (1.11)	0.19(1.24)
Antidepressants group × time			
Escitalopram		0	0
Duloxetine		−0.64 (3.1)	1.62 (3.47)
Sertraline		3.77 (1.81)*	3.75 (2.02)*
Venlafaxine		0.02 (1.30)	−0.74(1.45)
Vortioxetine		1.82 (2.52)	−1.57(2.81)
Combined trazodone (no) × time		0.88 (0.88)	1.55 (0.98)

Values represent estimated effect sizes (β) and corresponding standard errors (SE);

*p < 0.05,

**p < 0.01,

***p < 0.001; PHQ-9, Patient Health Questionnaire-9; GAD-7, Generalized Anxiety Disorder Questionnaire-7.

**(2) Efficacy in cancer and non-cancer groups**

The PHQ-9 and GAD-7 scores were not different between cancer and non-cancer patients in the fix model (both *p* > 0.05). The cancer status group × time interaction effects remained insignificant after controlling for confounding factors (all *p* > 0.05).

**(3) Efficacy in antidepressants groups**

When comparing the four antidepressants with escitalopram, only the PHQ-9 scores in the venlafaxine group was lower (β = 1.63, SE = 0.74, *p* < 0.05). No significant differences were observed with other antidepressants compared to escitalopram. The antidepressants group × time interaction effects in the sertraline group became significantly less pronounced at week 4 for GAD-7 (β = 2.69, SE = 1.07, *p* < 0.05) score changes compared to escitalopram, and at week 8 for both GAD-7 (β = 3.77, SE = 1.81, *p* < 0.05) and PHQ-9 (β = 3.75, SE = 2.02, *p* < 0.05) score changes compared to escitalopram. When comparing antidepressants with and without trazodone, the group without trazodone showed significantly less pronounced PHQ-9 score changes at week 4 compared to the group with trazodone (4th-week PHQ-9 change: β = 2.06, SE = 0.74, *p* < 0.01). No significant differences were found in the trazodone group at the 8th-week time point.

#### 3.3.3 Cancer type-specific antidepressant response

A linear mixed model analysis was performed in the subgroup of patients with different cancer types, adjusting for the type of antidepressants and current cancer stage as confounding factors. The analysis also considered various treatment modalities, including surgery, radiotherapy, chemotherapy, immunotherapy, and endocrine therapy, to assess their impact on psychological outcomes (Supplementary Table 1). At baseline, patients in remission showed significantly lower PHQ-9 scores (β = −1.76, SE = 0.74, *p* < 0.05) compared to those undergoing treatment. And compared to breast cancer patients, patients with endocrine organ cancer had significantly higher GAD-7 (β = 2.49, SE = 1.09, *p* < 0.05) and PHQ-9 scores (β = 2.73, SE = 1.22, *p* < 0.05). Gynecological cancer patients had higher PHQ-9 scores (β = 2.61, SE = 1.06, *p* < 0.05). Hematologic malignancy patients showed significantly elevated PHQ-9 scores (β = 6.58, SE = 2.74, *p* < 0.05). Lung cancer patients had significantly higher PHQ-9 scores (β = 3.59, SE = 1.39, *p* < 0.05), while male reproductive/urinary system cancer patients had significantly elevated GAD-7 (β = 6.26, SE = 1.60, *p* < 0.001) and PHQ-9 scores (β = 5.93, SE = 1.79, *p* < 0.01). Interaction effects between cancer type and time indicated that gynecological cancer patients exhibited a significant reduction in PHQ-9 scores at week 4 (β = −5.84, SE = 2.42, *p* < 0.05) and in both PHQ-9 (β = −7.58, SE = 3.28, *p* < 0.05) and GAD-7 scores (β = −5.62, SE = 2.90, *p* < 0.05) at week 8, as compared to breast cancer patients. Radiotherapy had a significant effect, with patients not receiving it showing lower GAD-7 scores (β = −1.85, SE = 0.69, *p* < 0.01) at baseline. No significant effects were found for the other treatments on PHQ-9 or GAD-7 scores.

## 4 Discussion

Cancer patients are more susceptible to anxiety and depressive disorders, yet the management are often under-addressed, resulting in compromised quality of life. Given the significant heterogeneity in biological, environmental, and psychological factors underlying these conditions, the efficacy of a given antidepressant may differ in cancer patients compared to the general non-cancer population (Lynall and McIntosh, 2023). Therefore, in this study we found that the efficacy of antidepressants is comparable in cancer and non-cancer patients in treating anxiety and depressive disorders. Notably, the efficacy of escitalopram matches that of duloxetine, venlafaxine, and vortioxetine, each of which surpassed sertraline in cancer patients.

The retrospective cohort study included cancer and non-cancer patients receiving antidepressant treatments. The cancer group had approximately five times as many females as males, likely due to the prevalence of breast cancer or the higher susceptibility of females to depression and anxiety disorders, aligning with previous research (Parker and Brotchie, 2010). Cancer patients also experienced poorer sleep quality and a higher incidence of insomnia compared to non-cancer subjects, often treated with low doses of trazodone (≤ 50mg/kg) as an alternative to conventional sedatives to enhance sleep quality (Wichniak et al., 2017),. Currently, no standard antidepressant regimen is recommended for cancer patients with chronic anxiety and depression. In this study, escitalopram was the most frequently prescribed and used as the control, primarily due to its efficacy, tolerability, drug interactions, cost-effectiveness, and availability.

According to the monoamine hypothesis, second-generation antidepressants effectively alleviate depression and anxiety disorders by modulating the levels of serotonin, norepinephrine, dopamine, and other neurotransmitters (Krishnan and Nestler, 2008). However, previous reports on the effectiveness of antidepressants in cancer patients have been inconsistent. On the one hand, the ASCO guidelines did not include antidepressant treatments as the first-line approach for managing anxiety and depression in adult cancer patients (Andersen et al., 2023). Previous studies also suggested paroxetine and desipramine’s effects in breast cancer patients might be similar to a placebo (Musselman et al., 2006; Rutherford and Roose, 2013). On the other hand, another meta-analysis suggested that antidepressants can reduce the acute depressive symptoms of cancer patients, but only with limited quality of evidence (Vita et al., 2023). In this study, cancer patients showed significant improvements in anxiety and depression symptoms at the 4th and 8th weeks of follow-up, similar to non-cancer patients. Our findings support the standardized prescription of antidepressants for managing anxiety and depression in cancer patients.

Furthermore, escitalopram has been shown to be more effective than sertraline in the treatment of anxiety and depression. Another network meta-analysis including 21 antidepressants suggests that escitalopram might have the highest efficacy and tolerability in the general population (Cipriani et al., 2018). Another study on breast cancer survivors suggest that escitalopram can effectively alleviate hot flashes and depressive symptoms (Biglia et al., 2018). Our study was consistent with prior findings and confirmed the role of escitalopram in its efficacy in cancer patients.

Different cancer types exhibited varied responses to antidepressant treatments. In this study, breast cancer patients were selected as the control group due to their larger numbers. These individuals experience a broad spectrum of psychological and emotional trauma from various factors such as post-surgical body shape changes, lymphedema, fertility issues, and hormonal imbalances (Pilevarzadeh et al., 2019). Compared with the breast cancer patients, gynecological cancer patients showed higher initial depression scores but responded better to antidepressants, likely influenced by peri-menopausal changes post-oophorectomy. Despite a favorable prognosis, thyroid cancer survivors often face psychological distress and reduced quality of life, driven by factors like fear of recurrence, ongoing surveillance, and feelings of isolation (Dionisi-Vici et al., 2021). Similarly, hematologic malignancy patients have higher rates of depression, potentially linked to symptom burden, including fatigue, sleep disruption, and pain, as well as the fear of recurrence (Kuczmarski et al., 2024). Lung cancer, male reproductive/urinary system cancer patients also showed higher initial anxiety and depression scores, potentially linked to significant somatic symptoms: inflammatory cytokine disorders in lung cancer, and psychological issues related to sexual function, urinary complications, pain, and anhedonia in male reproductive/urinary system cancer (Watts et al., 2014; Sharpley et al., 2023; Mcfarland et al., 2020). Therefore, when assessing anxiety and depression in cancer patients, it is crucial to consider the unique characteristics of different cancer types to more accurately understand the underlying symptoms.

This study was conducted at a single hospital in China with a predominantly Chinese patient population, which may limit the generalizability of the findings to other healthcare settings and populations. Moreover, while the effectiveness assessment was based on self-reported survey data, incorporating objective medical parameters, such as brain imaging or hippocampal volume, could provide a deeper understanding of the neuropsychological impact on patients. Future multi-center studies that include diverse demographics and combine both subjective and medical assessments would help validate and expand upon these results.

## 5 Conclusion

In conclusion, our study suggest that antidepressants are equally effective in treating anxiety and depression in cancer patients as they are in the non-cancer population. Among five antidepressants, escitalopram equals duloxetine, venlafaxine, and vortioxetine, and may have a superior effect compared to sertraline. Patients with different types of cancer experience varying degrees of psychological symptoms. Of note, gynecological cancer patients may respond quickly to antidepressant treatments compared to breast cancer patients. Our study highlights the necessity of personalized psychiatric care in oncology management, advocating for antidepressant treatments tailored to specific cancer types and patient psychological profiles. Integrating comprehensive mental health strategies into cancer care can greatly enhance patient quality of life, potentially improving therapeutic outcomes and treatment adherence.

## Data Availability

The raw data supporting the conclusions of this article will be made available by the authors, without undue reservation.
