# Supplementary material for: Efficacy comparison of five antidepressants in treating anxiety and depression in cancer and non-cancer patients
Source: Front Neurosci. 2024 Oct 30;18:1485179. doi: 10.3389/fnins.2024.1485179 (PMC11557551; doi:10.3389/fnins.2024.1485179)
Supplement: Supplementary file 1 [file Table_1.docx]

Supplemental Table 1：Results of the mixed linear model of factors influencing PHQ-9 and GAD-7 in cancer patients

| **Variables** | **Categories** | **GAD-7 scores** | | **PHQ-9 scores** | |
| --- | --- | --- | --- | --- | --- |
| **Follow-up time** | Baseline | 0 | | 0 | |
|  | Week 4 | -6.59(2.81) ^*^ | | -6.75(3.18) ^*^ | |
|  | Week 8 | -6.03(3.06) ^*^ | | -6.14(3.46) | |
| **Antidepressants** | Escitalopram | 0 | | 0 | |
|  | Duloxetine | -1.12(1.67) | | -1.00(1.88) | |
|  | Sertraline | 1.18(1.27) | | 0.98(1.43) | |
|  | Venlafaxine | 0.91(1.03) | | 0.75(1.16) | |
|  | Vortioxetine | -1.56(2.79) | | 1.81(3.13) | |
| **Combined trazodone** | Yes | 0 | | 0 | |
|  | No | -0.49(0.57) | | -0.67(0.63) | |
| **Current Cancer Stage** | Undergoing Treatment | 0 | | 0 | |
|  | In Remission | -2.08(0.66) | | -1.76(0.74) ^*^ | |
|  | Cancer reoccurring | 1.31(1.02) | | 0.61(1.15) | |
| **Surgery** | Yes | 0 | | 0 | |
|  | No | -0.65(0.72) | | -0.29(0.81) | |
| **Radiotherapy** | Yes | 0 | | 0 | |
|  | No | -1.85(0.69) ^**^ | | -0.99(0.78) | |
| **Chemotherapy** | Yes | 0 | | 0 | |
|  | No | 0.79(0.64) | | 0.36(0.72) | |
| **Immunotherapy** | Yes | 0 | | 0 | |
|  | No | 1.02(1.66) | | 1.07(1.86) | |
| **Targeted therapy** | Yes | 0 | | 0 | |
|  | No | 0.14(0.94) | | -0.15(1.06) | |
| **Endocrine therapy** | Yes | 0 | | 0 | |
|  | No | 0.50(0.84) | | -0.59(0.94) | |
| **Types of cancer** | Breast cancer | 0 | | 0 | |
|  | Digestive system cancer | 0.76(0.89) | | 1.57(1.00) | |
|  | Endocrine organ cancer | 2.49(1.09) ^*^ | | 2.73(1.22) ^*^ | |
|  | gynecological cancer | 1.37(0.94) | | 2.61(1.06) ^*^ | |
|  | Head and neck cancer | 0.15(1.45) | | 1.04(1.63) | |
|  | Hematologic malignancy | 2.65(2.44) | | 6.38(2.74) ^*^ | |
|  | Lung cancer | 1.82(1.24) | | 3.59(1.39) ^*^ | |
|  | Soft tissue cancer | 1.57(2.43) | | 1.05(2.70) | |
|  | Male reproductive/urinary system cancer | 6.26(1.60) ^***^ | | 5.93(1.79) ^**^ | |
| **Interaction effects** (influencing factors × time) | |  | |  | |
| **Week 4 – baseline** | |  | |  | |
| **Surgery(no)** **× time** | | 1.22(1.23) | | 2.16(1.40) | |
| **Radiotherapy(no) × time** | | 1.43(1.29) | | 0.43(1.46) | |
| **Chemotherapy(no) × time** | | -2.13(1.20) | | -2.50(1.36) | |
| **Immunotherapy(no) × time** | | 1.49(2.65) | | -0.57(3.00) | |
| **Targeted therapy(no) × time** | | -1.13(1.68) | | 1.21(1.90) | |
| **Endocrine therapy(no) × time** | | -0.68(1.56) | | 0.78(1.77) | |
| **Types of cancer × time** | |  | |  | |
|  | Breast cancer | 0 | 0 | |  |
|  | Digestive system cancer | -0.20(1.49) | 0.89(1.69) | |  |
|  | Endocrine organ cancer | 3.22(1.82) | 2.51(2.05) | |  |
|  | Gynecological cancer | -4.21(2.14) | -5.84(2.42) ^*^ | |  |
|  | Head and neck cancer | --0.21(2.41) | -1.56(2.73) | |  |
|  | Hematologic malignancy | -4.36(3.74) | -4.68(2.71) | |  |
|  | Lung cancer | -4.39(2.45) | -3.12(2.77) | |  |
|  | Soft tissue cancer | 1.04(3.08) | 0.54(3.49) | |  |
|  | Male reproductive/urinary ystem cancer | -3.31(2.62) | -3.42(2.96) | |  |
| **Week 8 – baseline** | |  | |  | |
| **Surgery(no) × time** | | -1.55(1.73) | | 0.74(1.96) | |
| **Radiotherapy(no) × time** | | -0.36(1.64) | | -0.17(1.85) | |
| **Chemotherapy(no) × time** | | -0.82(1.67) | | -0.30(1.89) | |
| **Immunotherapy(no) × time** | | 0.02(3.03) | | -0.60(3.43) | |
| **Targeted therapy(no) × time** | | 0.55(2.00) | | -0.46(2.26) | |
| **Endocrine therapy(no) × time** | | -1.70(1.85) | | -1.37(2.10) | |
| **Types of cancer × time** | |  | |  | |
|  | Breast cancer | 0 | | 0 | |
|  | Digestive system cancer | -1.31(2.14) | | 0.85(2.42) | |
|  | Endocrine organ cancer | -1.39(2.35) | | -2.63(2.66) | |
|  | Gynecological cancer | -5.62(2.90) ^*^ | | -7.58(3.28) ^*^ | |
|  | Head and neck cancer | 0.26(3.43) | | 3.79(3.88) | |
|  | Lung cancer | 0.61(2.58) | | -3.00(2.91) | |
|  | Soft tissue cancer | 0.44(4.76) | | 1.28(5.38) | |

Values represent estimated effect sizes (β) and corresponding standard errors (SE); *p<0.05, **p<0.01, *** p<0.001; PHQ-9: Patient Health Questionnaire-9; GAD-7: Generalized Anxiety Disorder Questionnaire-7.
